# The effect of the lamin A and its mutants on nuclear structure, cell proliferation, protein stability, and mobility in embryonic cells

**DOI:** 10.1007/s00412-016-0610-9

**Published:** 2016-08-17

**Authors:** Katarzyna Piekarowicz, Magdalena Machowska, Ewelina Dratkiewicz, Daria Lorek, Agnieszka Madej-Pilarczyk, Ryszard Rzepecki

**Affiliations:** 1 Laboratory of Nuclear Proteins, Faculty of Biotechnology, University of Wroclaw, Fryderyka Joliot-Curie 14a, 50-383 Wroclaw, Poland; 2 Neuromuscular Unit, Mossakowski Medical Research Centre, Polish Academy of Sciences, Pawinskiego 5, 02-106 Warsaw, Poland

**Keywords:** Nuclear envelope, Lamin A/C, Human embryonic kidney 293, Laminopathy, Emery-Dreifuss muscular dystrophy, Hutchinson-Gilford progeria syndrome

## Abstract

*LMNA* gene encodes for nuclear intermediate filament proteins lamin A/C. Mutations in this gene lead to a spectrum of genetic disorders, collectively referred to as laminopathies. Lamin A/C are widely expressed in most differentiated somatic cells but not in early embryos and some undifferentiated cells. To investigate the role of lamin A/C in cell phenotype maintenance and differentiation, which could be a determinant of the pathogenesis of laminopathies, we examined the role played by exogenous lamin A and its mutants in differentiated cell lines (HeLa, NHDF) and less-differentiated HEK 293 cells. We introduced exogenous wild-type and mutated (H222P, L263P, E358K D446V, and ∆50) lamin A into different cell types and analyzed proteins’ impact on proliferation, protein mobility, and endogenous nuclear envelope protein distribution. The mutants give rise to a broad spectrum of nuclear phenotypes and relocate lamin C. The mutations ∆50 and D446V enhance proliferation in comparison to wild-type lamin A and control cells, but no changes in exogenous protein mobility measured by FRAP were observed. Interestingly, although transcripts for lamins A and C are at similar level in HEK 293 cells, only lamin C protein is detected in western blots. Also, exogenous lamin A and its mutants, when expressed in HEK 293 cells underwent posttranscriptional processing. Overall, our results provide new insight into the maintenance of lamin A in less-differentiated cells. Embryonic cells are very sensitive to lamin A imbalance, and its upregulation disturbs lamin C, which may influence gene expression and many regulatory pathways.

**Electronic supplementary material:**

The online version of this article (doi:10.1007/s00412-016-0610-9) contains supplementary material, which is available to authorized users.

## Introduction

Mutations in genes encoding for lamins and lamina-associated proteins, such as LEM domain proteins (LAP2, emerin, and MAN1), cause human genetic disorders that are collectively referred to as laminopathies or envelopathies. This heterogeneous group comprises diseases of different clinical course, severity, and molecular background. The best-known laminopathy phenotypes are based on mutations in the *LMNA* gene (autosomal), which encodes for lamins A and C, and the *EMD* gene (X-linked), which encodes for emerin (Worman and Bonne 2007; Zaremba-Czogalla et al. 2011).

Tissues of mesenchymal origin are affected in these disorders and the phenotypic subgroups include muscular, peripheral neurogenic, lipodystrophy, and premature aging syndromes (Worman and Bonne 2007). The most common disease phenotypes are the “classical,” muscle-related laminopathies, such as Emery-Dreifuss muscular dystrophy type 2 (EDMD2) (Bonne et al. 1999), with symptoms such as muscle contractures, generalized muscle atrophy, rigidity of the spine, cardiac insufficiency, and ventricular arrhythmia. One of the most severe genetic disorders from this group is the very rare Hutchison-Gilford progeria syndrome (HGPS). Its typical cause is a 1824C>T mutation in the *LMNA* gene, resulting in the activation of a cryptic splicing site in exon 11 of the primary transcript (Eriksson et al. 2003). This leads to the synthesis of a lamin A deletion mutant protein (lamin A ∆50, progerin) lacking 50 amino acids. The mutation prevents the last step of prelamin A posttranslational modification, meaning the protein remains permanently farnesylated.

Various disease phenotypes arise due to the modulation of different intracellular processes by lamin A/C, including intracellular signaling, regulation of transcription, maintenance of nuclear shape, chromatin organization, and nuclear pore spacing (Wiesel et al. 2008; Shimi et al. 2010; Dubinska-Magiera et al. 2013). Thus, mutations in *LMNA* gene, depending on their location and type, may disturb different functions of lamin A/C and affect various processes. The interactions of lamin A with LAP2α affect on the pRb signaling pathway, which is involved in proliferation and regeneration, so there is a high probability that a major mechanism in many of the diseases is this pathway (Markiewicz et al. 2002; Pekovic et al. 2007; Cohen et al. 2013).

Hundreds of mutations in the *LMNA* gene have been described in patients. The related clinical courses have various onsets, phenotypes, and severities. The mutations can be seen in the Universal Mutation Database (http://www.umd.be), the Human Intermediate Filament Database (http://www.interfil.org), and the Leiden Open Variation Database (http://www.dmd.nl). Some mutations, especially the first that had been identified, have been thoroughly described and analyzed using various model systems, such as patients’ fibroblasts and myoblasts, cells transfected with constructs encoding for mutated lamin A, transgenic animals, and cells obtained from them. Each model system offers a number of possibilities to dissect the various molecular mechanisms that give rise to the phenotype associated with particular mutations. The limiting factors on such studies are the restricted lifetime and availability of the primary cells, especially for non-skin cells.

Analyses of skin fibroblasts revealed abnormalities such as honeycomb and foci-forming expression patterns of lamin A and nuclear blebbing and lobulations that disturb other nuclear envelope (NE) proteins (Vigouroux et al. 2001; Favreau 2003; Caux et al. 2003; Muchir et al. 2003). There are also a few mouse models with *LMNA* deletion variants (Azibani et al. 2014) and lamin A mutations: ∆K32 (Bertrand et al. 2012), H222P (Arimura et al. 2005), and N195K (Mounkes et al. 2005). Although a considerable amount of data was gathered using these models, the disease phenotypes in mice differ from those seen in humans.

Transfection of cell lines or primary cells allows the derivation of the broadest screening and equal genetic background for the comparison of mutants. Mouse embryonic fibroblasts obtained from *LMNA* ^*−/−*^ mice transfected with lamin A variants clearly showed that some mutants tend to aggregate and that emerin localization can be disturbed by specific mutations (Raharjo et al. 2001; Holt et al. 2003). The impact of exogenous lamin A mutants on cell phenotype was also investigated in the cell lines C2C12 (Ostlund et al. 2001; Scharner et al. 2011), H9C2 and COS7 (Sylvius et al. 2008), CHO (Broers 2004), and in HeLa cells (Hübner et al. 2006; Manju et al. 2006), neonatal rat cardiomyocytes (Arimura et al. 2013), human mesenchymal stem cells (Malashicheva et al. 2015), and human embryonic kidney 293 (HEK 293) cells (Tan et al. 2015; Liu et al. 2016). It was shown that lamin A mutants diminish proliferation capacity (colony formation) and enhance the differentiation ability of transduced mesenchymal stem cells (Malashicheva et al. 2015). Lamin A and its mutants R48P, R249W, I373V, and I497_E536del showed a tendency to aggregate in HEK 293 cells transfected with constructs encoding these proteins (Tan et al. 2015). The increased mobility of the lamin A mutant R386K in a nucleoplasmic location was observed in bleaching experiments in CHO cells (Broers et al. 2005). Recent studies demonstrated that some mutations may lead to aggregation of lamins, thus contributing to the disease phenotype (Alastalo et al. 2015; West et al. 2016). Systemic screening of pathological lamin A interaction was performed using yeast two-hybrid system and partially verified by pull-down analyses, using 89 lamin A mutations in U2OS cell line. This study confirmed the role of Ig fold in interactions and confirmed the tissue-specific loss of interaction as a main molecular background of the disease phenotype (Dittmer et al. 2014).

In somatic tissues, the *LMNA* gene encodes for lamins A and C, due to alternative splicing. Although they share many functions, they have different expression patterns, regulatory mechanisms, or assembly properties in different tissues (Al-Saaidi and Bross 2015). The targeting of lamin C and some other proteins (e.g., emerin) to the NE depends on lamin A localization (Pugh et al. 1997; Vaughan et al. 2001), but it is not clear how lamin mutations influence this process. Low-level expression of mutant proteins did not influence lamin C distribution in C2C12 cells (Scharner et al. 2011), but in COS-7 cells, lamin C was slightly diminished in the nuclear rim due to the presence of some lamin A mutants (Motsch et al. 2005).

Studies investigating different expression levels and cell types were generally performed using antibodies recognizing both lamins A and C, so it was not possible to clarify if lamin C distribution was affected by lamin A mutations (Ostlund et al. 2001; Vigouroux et al. 2001; Muchir et al. 2004). Therefore, it is not clear whether lamin C can maintain its function in the presence of lamin A mutant protein or indeed compensate for the loss of lamin A function.

**Fig. 1:**
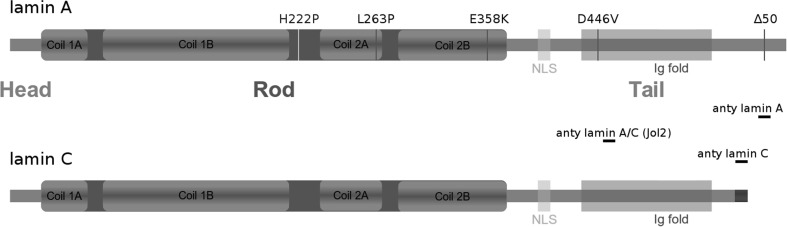
A scheme of lamins A and C indicating the location of the analyzed mutations within their domains and position of epitopes recognized by anti lamin A/C antibodies used in this study (Machowska et al. 2015)

Our study focuses on two previously uncharacterized mutations in the *LMNA* gene: L263P and D446V (Fig. 1, Table 1 for references). They had been discovered in Polish patients with EDMD2. We also used three other mutations with different molecular properties: H222P, E358K, and ∆50 (Fig. 1, Table 1). Reports on L263P and D446V were published, but they have yet to be studied in tissue culture or animal model systems. Patients bearing such *LMNA* gene mutations show phenotypes corresponding to muscle-related laminopathies (see Table 1).

**Table 1:** Clinical characterization of the patients in the study

Mutation	Clinical characteristics	References
c.665A>C p.His222Pro p.H222P	EDMD2 (familial) Initial symptoms: Achilles tendon and elbow contractures Clinical presentation: Scapular wasting; contractures of elbows and ankles; rigidity of the spine; weakness of proximal and distal muscles Cardiac arrhythmia: Yes	(Bonne et al. 2000)
c.788T>C p.Leu263Pro p.L263P	EDMD2 phenotype (familial) Initial symptoms: Elbow contractures (from age of 11). Clinical presentation: Atrophy of arms and calves; contractures of elbows and ankles; rigidity of the spine Cardiac arrhythmia: AVB 1/2/3, AF, VT, VF; pacemaker (aged 28); died at 29 due to decompensated cardiac insufficiency and ventricular arrhythmia	(Niebroj-Dobosz et al. 2008; Hausmanowa-Petrusewicz et al. 2009)
c.1072G>A p.Glu358Lys p.E358K	EDMD2 phenotype (sporadic) Initial symptoms: Elbow, knee, and ankle contractures (from age of 9). Clinical presentation: Requires wheelchair; generalized muscle atrophy; contractures of arms, elbows, knees and ankles; rigidity of the spine Cardiac arrhythmia: AVB 1/2/3, PAF, VT, VF; pacemaker (aged 25); cardiac insufficiency NYHA 2/3; episodes of cardiac and respiratory insufficiency; requires respiratory support at night	(Fidzianska and Glinka 2007; Niebroj-Dobosz et al. 2008; Hausmanowa-Petrusewicz et al. 2009)
	EDMD2 phenotype + mild polyneuropathy (familial) Initial symptoms: Proximal muscle weakness; slim and less agile than peers (from age of 10) Clinical presentation: Atrophy of arms, thighs, and calf muscles; weakness of proximal muscles; rigidity of the spine; slight elbow, knee and ankle contractures; decreased superficial sensation in distal parts of lower limbs Cardiac arrhythmia: SVT, SVEB, no pacemaker yet	(Madej-Pilarczyk et al. 2015)
c.1337A>T p.Asp446Val p.D446V	L-CMD/EDMD2 phenotype (sporadic) Initial symptoms: Elbow and ankle contractures (aged 6), rigidity of the spine (aged 7). Clinical presentation: Requires wheelchair; generalized muscle atrophy; contractures of arms, elbows, wrists and ankles; rigidity of the spine Cardiac arrhythmia: SVEB	(Niebroj-Dobosz et al. 2008; Hausmanowa-Petrusewicz et al. 2009; Madej-Pilarczyk et al. 2015)
c.1824C>T p.Gly608Gly p.G608G p.Δ50	HGPS Initial symptoms: Postnatal growth delay (aged 3 to 6 months) Clinical presentation: Senile appearance; thin, wrinkled skin with discolorations and scleroderma-like lesions; hair loss; gray hair; short stature; osteolysis; dysplasia of clavicles; coxa valga; cataract; myocardial infarction or cerebral stroke due to early atherosclerosis Ultrastructural abnormalities: Nuclear blebbing; thickening of the lamina; peripheral distribution of heterochromatin	(Eriksson et al. 2003; De Sandre-Giovannoli et al. 2003)

Mutations L263P, E358K, and D446V were identified in Polish patients. Positions of the mutations are marked on lamin A diagram (Fig. 1)

*SVT* supraventricular tachycardia, *VT* ventricular tachycardia, *VF* ventricular fibrillation, *SVEB* supraventricular ectopic beats, *AVB* atrioventricular block, *AF* atrial fibrillation, *PAF* paroxysmal atrial fibrillation

There are practical limitations on the availability of patient cells, so we conducted our studies on a tissue culture model system that used both transient transfection and stable cell lines. Three cell lines were used: normal human dermal fibroblast (NHDF), HeLa, and HEK 293. Most of the experiments were performed on HEK 293 cells as it is the best model for transient transfection studies due to the possibility of achieving high transfection efficiency. HEK 293 cells are usually neglected in such studies, which typically focus on differentiated cells with a high level of lamin proteins. Lamin A/C depletion was shown to affect embryonic cell differentiation (Sehgal et al. 2013), but the influence of dominant mutations was not previously investigated in this context. Lamins A and C are also critical for muscle cell differentiation (Frock et al. 2006). Since they affect intracellular signaling, understanding the effect of mutated lamin A expression on cells that are not fully differentiated may help to understand its function during development.

In this paper, we demonstrate the subcellular distribution of lamin A mutants and the effect of particular mutations on the distribution and expression of endogenous NE proteins. We also report on analyses of the proliferation of cells bearing a particular mutation and on the mobility of mutated lamin A proteins. Additionally, we proposed a mechanism for lamin A removal in HEK 293 cells.

## Materials and methods

### Constructs and directed mutagenesis

Professor C.J. Hutchison’s lab supplied the N-terminal fusion protein prelamin A in plasmid pEGFP-C1 (Clonetech). Mutations were introduced via site-directed mutagenesis using the following primer pairs for PCR:

- H222P: 5′-accaagcgccgtcctgagacccgactg-3′, 5′-cagtcgggtctcaggacggcgcttggt-3′
- L263P: 5′-agcagtataagaaggagccggagaagacttattctgc-3′; 5′-gcagaataagtcttctccggctccttcttatactgct-3′
- E358K: 5′-cagcagcagctggacaagtaccaggagcttc-3′, 5′-gaagctcctggtacttgtccagctgctgctg-3′
- D446V: 5′-gtggaggaggtggttgaggagggcaag-3′, 5′-cttgccctcctcaaccacctcctccac-3′

All constructs were verified via sequencing with the following primers: For 5′-atggtcctgctggagttc-3′; Rev 5′-tacaaatgtggtatggctg-3′. The progerin construct with lamin A lacking 50 amino acids (pEGFP-lamin A ∆50) was donated by Professor Tom Misteli (Addgene plasmid #17653) (Scaffidi and Misteli 2005).

### Cell culture

HEK 293 cells were cultured in Dulbecco’s modified Eagle medium with high glucose (DMEM 4.5 g/l glucose, Lonza). The HeLa and NHDF cells were cultured in minimum essential medium alpha (MEMα, Lonza). All media were supplemented with 10 % fetal bovine serum (Sigma), GlutaMAX supplement (Gibco), antibiotics, and antimycotics (Gibco). The HEK 293 and HeLa cell lines were purchased from the Cell Line Collection of the Polish Academy of Sciences, Institute of Immunology and Experimental Therapy in Wroclaw. The NHDF cell line was purchased from Lonza.

### Cell transfection

HEK 293, HeLa, and NHDF cells were plated on a 24-well plate 24 h before transfection. For western blot analysis, cells were transfected using Turbofect Reagent (Thermo Scientific) according to the manufacturer’s instructions. For other purposes, HEK 293 cells were transfected via electroporation with a Lonza CLB-Transfection Device according to the manufacturer’s instructions. For immunofluorescence analysis, the cells were seeded on glass coverslips after electroporation. Transfection efficiency was routinely ∼90 % for HEK 293, ∼50 % for HeLa, and ∼20 % for NHDF.

The plasmid pEGFP-C1 was used with the following cDNAs: EGFP, EGFP-lamin A, and EGFP-lamin A ∆50 (progerin); and the following EGFP-lamin A mutants: H222P, L263P, E358K, and D446V. Four hours after transfection with Turbofect or 18 h after electroporation, the medium was replaced with fresh complete medium. Cells were monitored using an Axiovert 40 CFL fluorescence microscope. After an appropriate time, the cells were fixed for immunofluorescence experiments or collected by trypsinization for western blot experiments.

### Selection of stable transgenic cell lines

After HEK 293 transfection, single cells were transferred to fresh wells. Clones were verified for proper lamin A localization using fluorescent microscopy. For EGFP, EGFP-lamin A, and the least toxic mutants ∆50 and D446V sublines were established and cultivated with antibiotic G418 (Sigma) for over 1 month before analysis.

### Electrophoresis and western blot analysis

Whole cell extracts were lysed in Laemmli buffer, separated via SDS-PAGE (usually with 10 % gels), transferred to nitrocellulose membranes, and blocked in 5 % non-fat milk in PBS for 1 h. Primary antibodies were applied overnight in 4 °C, while secondary antibodies conjugated with horseradish peroxidase (HRP) were applied for 1 h at room temperature. Proteins were visualized using ECL substrate (BioRad). The primary antibodies were as follows: mouse anti-human lamin A/C (Jol2, 1:500), mouse anti-human lamin A (1:1000, Millipore), mouse anti-human β-tubulin (1:3000, Sigma), rabbit anti-human emerin prepared in our lab against an N-terminal fragment of human emerin (1:1000) (Rzepecki et al. 1998), and rabbit anti-human lamin C (1:500, donated by Professor C.J. Hutchison), while the secondary antibodies were donkey anti-mouse HRP (1:3000, Santa Cruz Biotechnology) and goat anti-rabbit HRP (1:20,000, Jackson ImmunoResearch).

### Indirect immunofluorescence

Cells were plated on glass coverslips, fixed in 4 % paraformaldehyde for 20 min, permeabilized with 0.5 % Triton X-100 in PBS for 5 min in 4 °C and washed with PBS. Fixed cells were incubated in primary antibody solution overnight at 4 °C, washed with PBS, and incubated for 1 h with secondary antibody solution at room temperature and washed again in PBS and water. Coverslips were mounted on glass slides using DABCO mounting medium (Fluka) with DAPI. Staining was visualized on a Zeiss 510 Meta confocal microscope using the 63X objective. Images were processed with ImageJ and Zeiss ZEN 2008 software for adjusting contrast, brightness, and size.

All antibodies were diluted in 1 % FBS in PBS. The primary antibodies used for staining were mouse anti-lamin A/C (Jol2, 1:20), rabbit anti-emerin (1:30, Proteintech), and rabbit anti lamin C (C1, 1:20, donated by Professor C.J. Hutchison). The secondary antibodies were donkey anti-mouse conjugated with TRITC (1:50), donkey anti-rabbit conjugated with Cy5 (1:50), and donkey anti-mouse conjugated with Daylight 649 (1:100), all purchased from Jackson ImmunoResearch.

### Quantitative RT-PCR

Total RNA was isolated from the cultured cells with the GeneMATRIX Universal RNA Purification Kit (EURx) and reverse-transcribed using Thermo Scientific Maxima Reverse Transcriptase (5 μg RNA per reaction). During isolation, the RNA samples were digested with DNase and then tested for the presence of genomic DNA using PCR.

The messenger RNA (mRNA) levels of lamins A and C were measured using specific primers (lamin A: 5′-taccgacctggtgtggaagg-3′, 5′-gagcgcaggttgtactcagc-3′; lamin C: 5′-taccgacctggtgtggaagg-3′, 5′-cggcggctaccactcacg-3′). Real-time PCR was performed in a 15-μl reaction volume that included 3 μl of 10× diluted template cDNA and SensiFAST SYBR No-ROX Kit (Bioline). Runs were performed in triplicate. The standard curve was prepared with plasmid containing cloned amplicons for both lamin A and C, synthesized by GeneCust. The plasmid dilution range was 7.5 ng to 7.5 fg of DNA and the amplification for the standard curve was always performed on the same 96-well plates as the samples and controls.

The reaction was performed with a CFX 96-Connect Real-Time PCR Detection System (Bio-Rad) with the following parameters: 95 °C for 2 min, followed by 40 cycles of denaturing at 95 °C for 5 s, annealing at 64 °C for 15 s, and extension at 72 °C for 8 s. The expression of lamins A and C was compared to the standard curve with known absolute copy numbers and calculated against 1 ng of RNA.

### Proliferation tests

HEK 293 sublines were seeded on 96-well plates in multiples. Cells were fixed after 5 days using 10 % TCA for 1 h at 4 °C, washed with water, and dried. Then, protein was stained with 0.4 % sulphorhodamine B (SRB) in 1 % acetic acid for 30 min at room temperature, washed with 1 % acetic acid, and dried. Protein-bound dye was dissolved in 150 μl 10 mM Tris base solution. The OD was measured at 500 nm using a microplate reader. The test was also validated: the cell lines were seeded on a 24-well plate, and cells were counted manually after 3 days.

### FRAP measurements

Fluorescence recovery after photobleaching (FRAP) analyses were performed on stable transfected HEK 293 sublines. Cells were cultured on glass coverslips and analyzed under a ×63 (water immersion) objective in culture medium using a Zeiss LSM 510 Meta microscope according to the manufacturer’s instructions. Optimal conditions (temperature, humidity, and CO_2_ levels) were maintained during analysis. For each subline, FRAP analyses were performed 4–10 times for up to 2 min per region. For the calculations, at least three measurements of the best quality from each set were selected. FRAP analysis software (Zeiss LSM 510 software – ZEN 2008) was used for the experimental setup and data collection (pre-bleach imaging, laser pulse, recovery imaging, collection of data, etc.).

## Results

### HEK 293 cells have very low lamin A protein expression levels

**Fig. 2:**
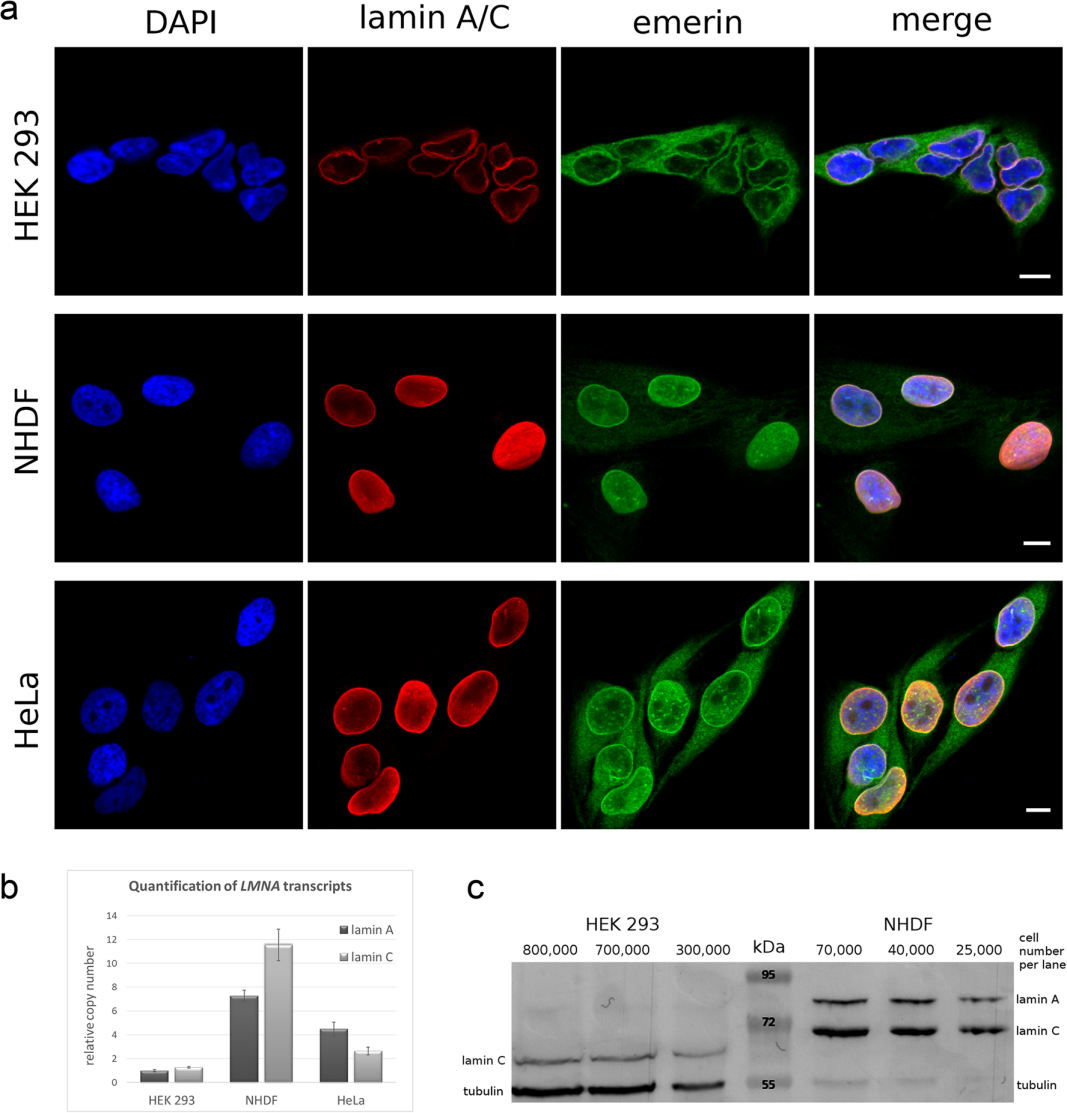
A characterization of the HEK 293, NHDF, and HeLa cell lines. **a** Lamins A and C were located within the NE and nucleoplasm of all of cell types, but their level was lowest in HEK 293 cells. Emerin localization differed between cell lines: in NHDF, it is located mostly in the nucleus; in HEK 293, it is mostly outside the nucleus; and in HeLa, it is mostly within the nuclear rim. The *scale bar* is 10 μm long. **b** A comparison of the mRNA levels for HEK 293, NHDF, and HeLa cells, measured separately for spliced forms of lamin A and C, performed using absolute quantitative RT-PCR. HEK 293 cells had the lowest levels of lamins A and C, and they were found to be similar. **c** Western blot analysis of protein extracts from HEK 293 and NHDF cells. The primary antibody recognizing the epitope common for lamins A and C was used. For NHDFs, the lamin A and C levels correspond to the mRNA level. For HEK 293 cells, the level of lamin C corresponded to the mRNA level, but the level of lamin A is much lower than expected from mRNA measurement. No signal was detected for HEK 293 lamin A, although there was ten times more cells per lane than for NHDFs. In addition, lamin C migrated faster for HEK 293 cells than for NHDFs. HeLa lamin C migration was the same as for NHDF lamin C (data not shown)

We initially decided to use HeLa, NHDF, and HEK 293 cells as the model system in our investigation of the mechanisms of pathogenesis of novel laminopathy mutations that are as yet uncharacterized. However, we focused the majority of our detailed experiments on HEK 293 cells, as they are relatively easy to transfect with high efficiency and have much better tolerance for overexpression than HeLa cells and fibroblasts (Thomas and Smart 2005). HEK 293 is an embryonic cell line, so it can also serve as a model for cells that are less differentiated (or not fully committed). This enabled us to look at the effect of lamin mutations on the properties of such cells. The effect of expression of particular lamin A mutants in cells that are in transition between embryonic and differentiating states may be important for the understanding of phenotype development. HEK 293 cells are a good example of such cells. All three cell lines express lamins and emerin, albeit at different levels and localizations (Fig. 2). In HEK 293 emerin was more cytoplasmic, while it was mostly in NE in HeLa cells (Fig. 2). In NHDFs, emerin is mostly in the nucleoplasm and at the NE. This was observed by immunofluorescence staining (Fig. 2a).

Western blot analysis with comparison to HeLa and NHDF cells showed that HEK 293 cells contain an undetectable amount of lamin A protein (Fig. 2c), as could be expected for less differentiated cells (Constantinescu et al. 2006). The general rule seems to be that stem cells have no or very low level of lamin A. However, it was shown that lamin A upstream promoter is active during very early development and then is downregulated (Kubben et al. 2011; Eckersley-Maslin et al. 2013). The level of lamin C in HEK 293 cells is low but at a clearly detectable level. Quantitative RT-PCR studies indicated that the amount of lamin A and C transcripts was, respectively, about 4.7 and 2.3 times lower than in HeLa cells and 7.6 and 9.9 times lower than in NHDFs (Fig. 2b). Surprisingly, in HEK 293 cells, the transcript’s level for lamin A was similar to that for lamin C. This discrepancy between the similar level of transcripts for lamins A and C and lack of detectable lamin A protein may suggest that there should be an additional, posttranscriptional mechanism decreasing the lamin A level. Since the lamin C protein level corresponds roughly to the level of its transcript (comparing the ratio between the protein and its transcript in all three cell lines, Fig. 2c), we may speculate that the mechanism is specific to lamin A.

Interestingly, western blot showed that the electrophoretic mobility of lamin C in HEK 293 cells was significantly higher than in NHDF (Fig. 2c) and HeLa cells, suggesting posttranslational modifications (proteolysis, dephosphorylation) or alternative splicing of lamin C. The very low level of lamin A and moderate level of lamin C may confirm the not fully differentiated status of HEK 293 cells.

### Subcellular distribution of lamin A mutants in transient transfection studies

In order to analyze particular lamin A mutants, we prepared a set of plasmids encoding fusion protein EGFP and prelamin A with introduced laminopathy-associated single base mutations (see Table 1 for the patient phenotype descriptions). A fluorescent tag was chosen mainly due to the technical requirements of FRAP measurements, and it was N-terminal to ensure proper posttranslational modification of prelamin A (Coffinier et al. 2010). The mutations were H222P, L263P, E358K, D446V, and ∆50, with EGFP and wild-type lamin A constructs used as controls.

We performed transfection of HEK 293 cells and examined them 24 h (shortest time lap allowing the observation of EGFP-tagged proteins) and 72 h (longest possible incubation without cell passaging, due to overgrowth) after transfection. Subsequently transfections of NHDFs and HeLa cells were also performed as comparable analyses for differentiated cells and as a reference point for comparison with other published studies (Figs. 5 and S1).

**Fig. 3:**
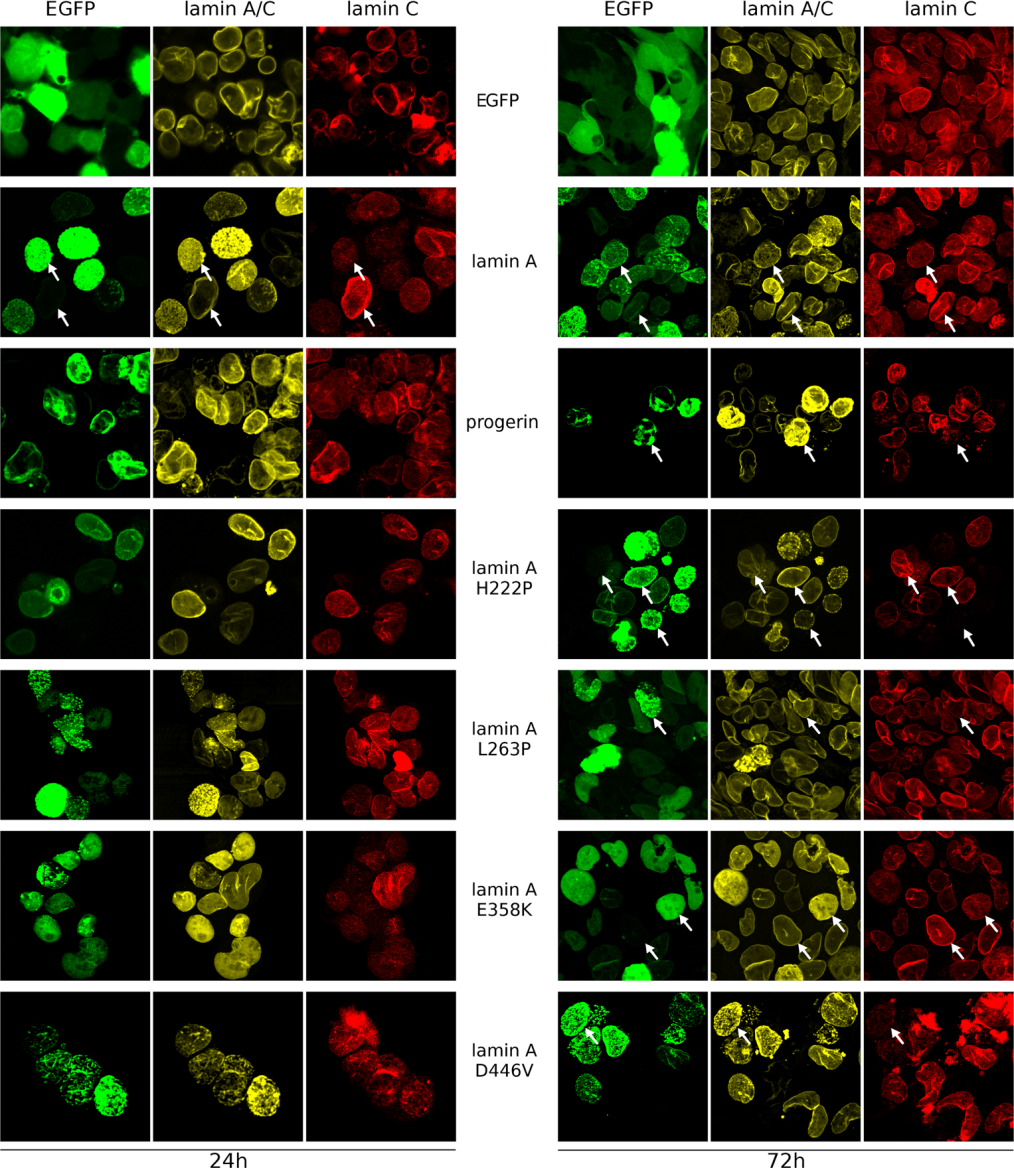
HEK 293 transfected with plasmids encoding EGFP, fusion protein EGFP-prelamin A, and its mutants, 24 h (*left panel*) and 72 h (*right panel*) posttransfection. Cells with different levels of exogenous lamin A proteins were typically found. There was a broad spectrum of phenotypes, depending on both mutation type and protein level. *Arrows* indicates cells with different levels of exogenous wild-type lamin A showing that the higher the lamin A level, the lower the lamin C level

Figure 3 shows typical phenotypes of transfected cells expressing lamin A and its mutants after 24 and 72 h. Each mutant has its own individual phenotype, which was typically stronger after 72 h, with the exception of D446V, the phenotype of which grew weaker over time. In the case of E358K, no significant changes in distribution were observed between 24 and 72 h. A similar protein distribution as in HEK 293 cells was also observed in NHDF (Fig. S1A) and HeLa cells (data not shown). In general, a low level of expression generated a typical NE distribution for most mutants, while a higher level of expression led to accumulation of exogenous lamin A in nucleoplasmic granules. The only exception was lamin A ∆50, which accumulates predominantly at the NE structures, frequently forming large, thick NE membrane stacks (see also Fig. S2).

### Lamin A mutants display different locations and properties

**Fig. 4:**
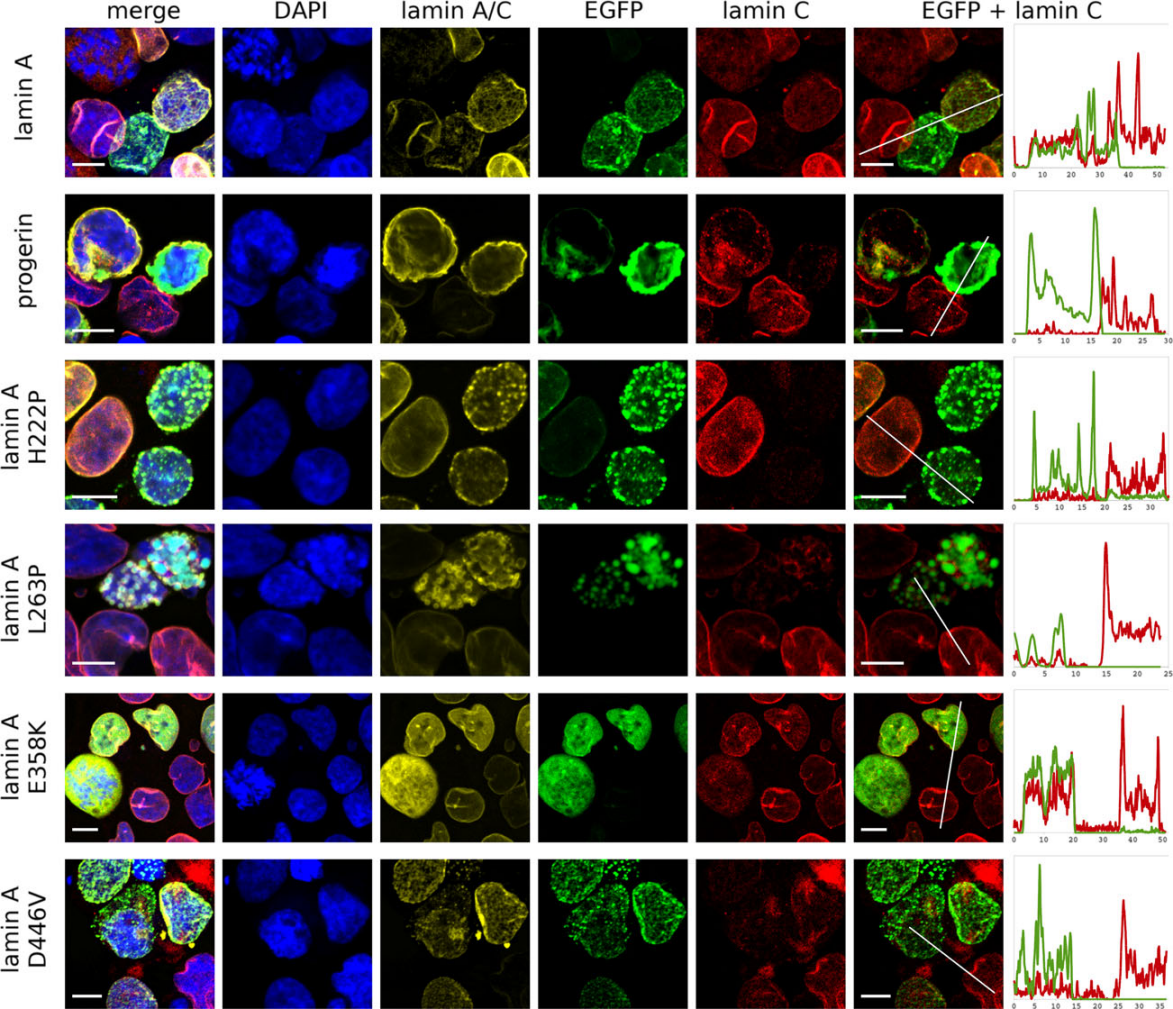
HEK 293 transfected with plasmids encoding EGFP, fusion protein EGFP-prelamin A, and its mutants, 72 h posttransfection. The *scale bar* is 10 μm long. The images show representative nuclei for each mutant and for comparison, at the same field of view, there is a control nucleus (not transfected cell). The *far right column* shows signal intensity as a function of distance [nm] along drown line for EGFP (*green*) and lamin C (*red*) channels with transfected cell first and control cell afterward

Figures 3 and 4 show that, depending on the expression level of a particular protein, different sets of morphological phenotypes can be observed at high magnification for both wild-type lamin A and its mutants. At low expression levels, exogenous lamin A was localized within the NE, usually forming a mesh that is clearly visible with confocal fluorescence microscopy. A high level of wild-type lamin A expression resulted in the additional appearance of lattice-like intranuclear aggregates of protein (see also Fig. S2).

The lamin A ∆50 mutant was solely localized in NE, as expected. A higher level of protein caused stronger envelope deformation (lobulations, invaginations, and membrane stacks) and abnormal NE thickening, but no EGFP signal was detected in the nucleoplasm (Fig. S2). The lamin A mutants H222P and L263P tended to aggregate and have speckle-like distributions, but L263P localized mostly in the nucleoplasm and H222P mostly within the NE. Deposits were more likely to be formed by L263P, even in cells with a relatively low level of exogenous protein. When protein foci grew bigger, L263P protein recruited endogenous lamin C into the foci structures. This mutant was very toxic for cells, especially with higher levels of protein. Most of the high-level mutant-expressing cells did not survive 72-h incubation after transfection. E358K showed a dispersed nucleoplasmic location with infrequent nucleoplasmic granules at high expression levels. D446V had the most similar distribution to wild-type lamin A. It formed a mesh, although with bigger aggregates than wild-type lamin A. After 72 h, the mutant protein was mostly dispersed into small intranuclear granules within the NE location. This protein also had a relatively low toxicity, so it was possible to establish a stable cell line encoding lamin A with this mutation.

Overall, the data showed that stronger phenotypes develop over time (or when protein expression is higher) and suggested that lamin C levels reduced or even disappeared completely at the NE and nucleus over time, regardless of the lamin A variant expressed.

### Exogenous lamins cause changes in the distribution but not the expression level of endogenous lamin C

Overexpression of all lamin A proteins (both wild-type and mutants) results in the reduction or even disappearance of lamin C in the cell nucleus and NE (as seen with immunofluorescence staining). This effect was stronger 72 h after transfection (Fig. 3). This effect is also clearly shown in Fig. 4. Distribution analyses performed on representative cells (Fig. 4) indicated that overexpression of exogenous lamin A and its mutants resulted in redistribution or disappearance of lamin C from cell nucleus of transfected cells. This effect was the lowest in the case of E358K (Figs. 3 and 4). Of the analyzed mutants, only E358K causes obvious redistribution of lamin C from the NE to nucleoplasm, even just 24 h after transfection. However, subsequently, lamin C seems to partially recover after 72 h in some cells (Fig. 3).

**Fig. 5:**
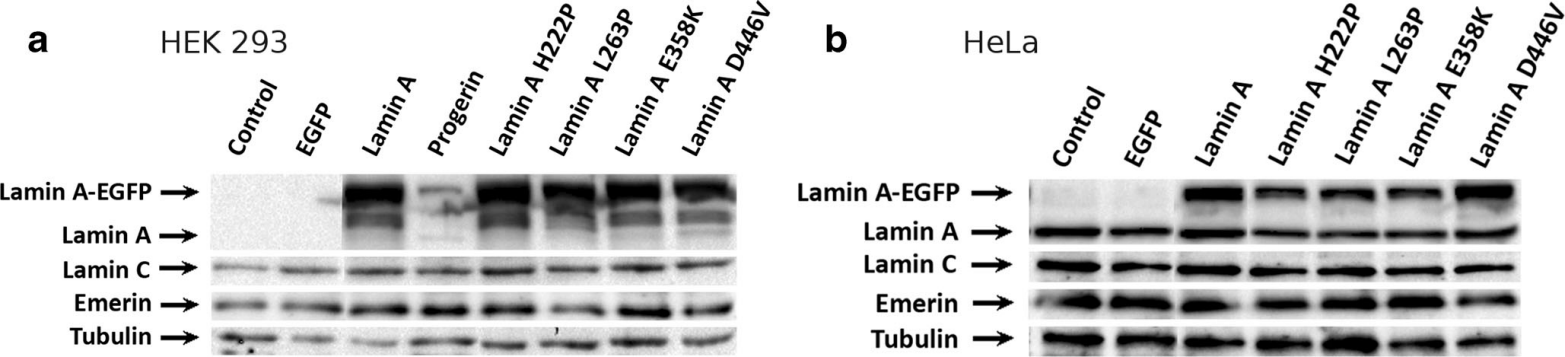
Western blot analysis of HEK 293 (**a**) and HeLa (**b**) cells, collected 72 h posttransfection with plasmids encoding EGFP, EGFP-prelamin A, and its mutants. Transfection efficiency was around 90 % for HEK 293 cells and around 50 % for HeLa cells. Exogenous lamin A underwent degradation in HEK 293 cells, but not in HeLa cells. Progerin detection was impaired due to the epitope not being recognized by the anti-lamin A antibody

To focus on the lamin C signal disappearance in immunofluorescence studies in cells overexpressing the various lamin A proteins, we conducted western blot experiments. HEK 293 cells were transfected (with over 90 % efficiency) with different lamin A constructs and the levels of lamin A, lamin C, emerin, and exogenous protein were analyzed using western blots (Fig. 5a). As a control, the same experiments were performed on HeLa cells (Fig. 5b) and NHDFs (Fig. S1B).

It was observed that although the lamin C signal disappeared from the NE in the immunofluorescence analysis (Figs. 3 and 4), there were no visible changes in the overall level of lamin C protein in the transfected cells (Fig. 5a). This suggests that lamin C was redistributed, not repressed or fully degraded. The cells with the highest lamin A expression showed lamin C distributed within the nucleoplasm or even cytoplasm (Fig. 4). Seventy-two hours after transfection, some of the cells with lower level of overexpressed proteins recovered lamin C in NE (Figs. 3 and 4).

Western blot analyses also indicated that there were no detectable changes in the endogenous lamin A or lamin C level also in HeLa and NHDF cells expressing exogenous lamin A proteins (Figs. 5b and S1 B). It must be remembered that in HEK 293 cells, the endogenous lamin A level is below the detection level for western blot analyses in comparison with HeLa and NHDF cells (Fig. 5 and S1B).

In transfected HEK cells expressing high levels of wild-type lamin A and its mutants ∆50, H222P, and D446V, lamin C disappears from the NE and cell nucleus 72 h posttransfection (Figs. 3 and 4). There was no detectable redistribution of lamin C into exogenously expressed lamin A foci inside the nucleus (if present). Mild expression of all the above mentioned proteins seems to not affect the endogenous lamin C signal intensity and distribution.

The presence of the most toxic lamin A mutant L263P in HEK 293 cells induced nucleoplasmic redistribution of endogenous lamin C into areas surrounding the granules of mutant lamin A (Fig. 4). This relocation was observed only when large nucleoplasmic foci of the mutated protein had been formed. In fully committed cells such as NHDFs, L263P also induced redistribution of lamin C into nucleoplasmic foci (Fig. S1A).

Taking all these results into consideration, our hypothesis of relocation of endogenous lamin C from the NE as a result of the expression of wild-type lamin A and its mutants is supported by experimental data from immunofluorescence and western blot analyses for less differentiated cells (HEK 293) and fully differentiated cells (HeLa, NHDFs).

### Lamin A maintenance in HEK 293 cells

HEK 293 cells have a very low level of endogenous lamin A compared to HeLa and NHDF cells and compared to their lamin C level (Figs. 2c and S1B). As mentioned above, lamin A protein level does not correspond to the transcript level (Fig. 2) comparing with lamin C protein and both lamins and corresponding transcripts in HeLa and NHDF cells. We suggest lamin A specific posttranscriptional mechanism responsible for this discrepancy.

In HEK 293 cells overexpressing wild-type lamin A, we observed consistently additional, specific products migrating faster than fusion protein EGFP-lamin A (the strong protein bands below the band corresponding to the EGFP-lamin A fusion protein in Fig. 5a). Such products were present also when mutant lamins were expressed. In HeLa cells and NHDFs, no such products were detected (Figs. 5b and S1).

In samples from transfected HEK 293 cells, when overdeveloped, it is possible to detect products migrating as wild-type lamin A degradation products with cleaved-off EGFP from expressed fusion protein. Other than lamin A, EGFP fusion proteins, overexpressed in HEK cells did not show this effect (data not shown). This is consistent with our observations from HEK 293 stable cell lines selected after transfection. During the establishment of the cell lines overexpressing lamin A variants, only cells with the lowest level of lamin A survived. Additionally, some clones showed partial cytoplasmic EGFP-tag localization, identical with EGFP protein alone. This can be a degradation product formed by cutting fusion protein. Degradation products were only observed in HEK 293 cells and only for lamin A (and mutants), and only the lamin A protein level is not proportional to the measured level of its transcript. Therefore, this indicates efficient posttranscriptional regulation of the lamin A level in HEK 293 cells. Proteolysis of lamin A may play a role in this regulation.

### Overexpression of lamin A and mutants does not affect emerin expression and distribution

**Fig. 6:**
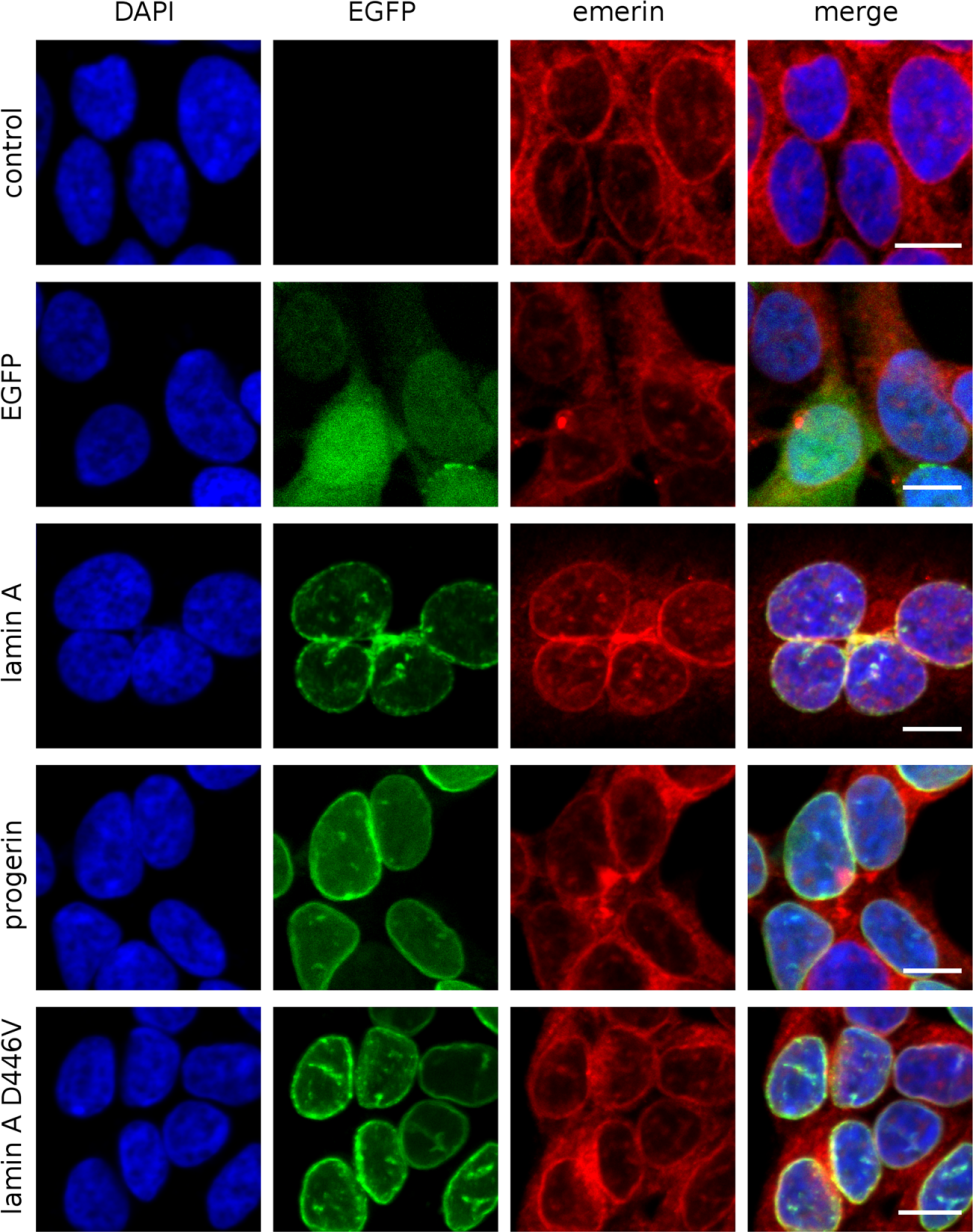
The location of endogenous emerin is not changed in HEK 293 sublines expressing EGFP, lamin A, progerin or lamin A D446 in comparison to control (not transfected cells). *Scale bar* is 10 μm long

We also analyzed the distribution of emerin in transfected cells since emerin recruitment into the nuclear envelope is dependent on lamin A. We were interested in detection of the potential effect of lamin A and mutant overexpression on emerin expression and distribution using transient transfection followed by western blot and immunofluorescence analyses. Western blot analyses indicated that there were no detectable changes in the emerin level in HEK 293, HeLa, and NHDF cells expressing exogenous lamin A proteins (Figs. 5 and S1B). Unfortunately, transient transfection studies were inconclusive, mostly due to the high heterogeneity of phenotypes and inconsistency of lamins’ expression level. Therefore, we used stable HEK 293 cell lines expressing lamin A and mutants for such analyses (Fig. 6). No changes in distribution of emerin were observed in stable cell lines expressing lamin A and mutants in HEK 293 cells.

### Lamin A mutants change the proliferation rate of HEK 293 cells

**Fig. 7:**
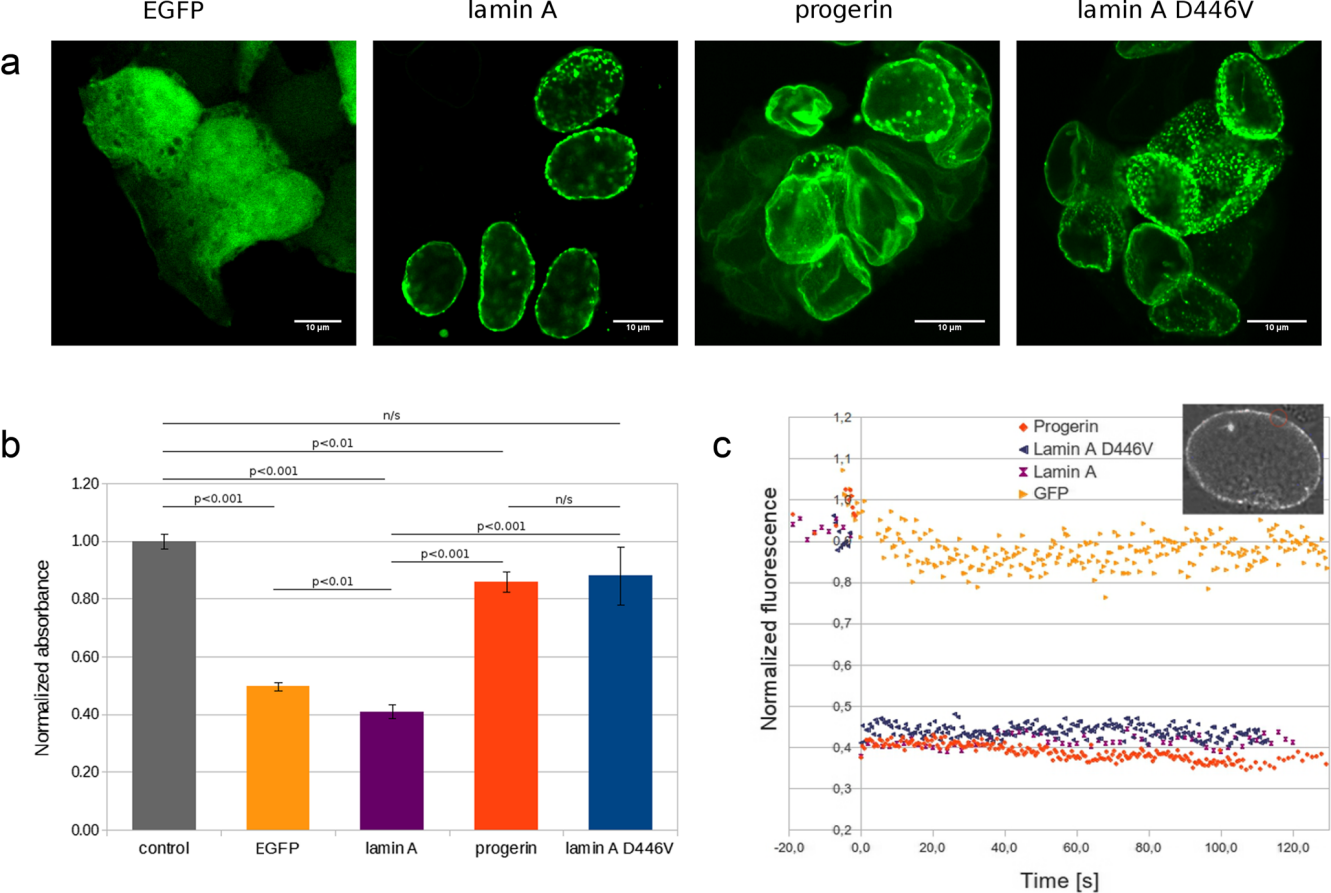
Analysis of HEK 293 sublines stably expressing EGFP alone and lamin A and its mutants D446V and Δ50 (progerin). **a** Confocal microscopy shows that all lamin A variants localize within the NE. Protein aggregates are much smaller than during transient transfection studies due to the lower level of exogenous proteins; however, some foci are still clearly visible. Progerin causes NE lobulations and the strongest deformation of the nuclear shape. **b** A proliferation comparison of the sublines was performed with the SRB assay 5 days after seeding. EGFP and lamin A expression significantly inhibits proliferation relative to the control, suggesting that the general effect of introducing exogenous protein may have a major role in this process. D446V and Δ50 abolished this effect, so they were able to increase the proliferation rate in embryonic cells. *n/s* not significant. **c** A comparison of protein mobility was performed using FRAP analysis. EGFP alone is very mobile: it immediately refilled the bleached area. By contrast, wild-type lamin A, progerin, and D446V proteins did not replace bleached lamin A. It shows that mutations did not significantly change protein mobility within the NE. Measurements were performed for up to 120 seconds. The *insert nucleus picture* shows the typical bleached area with a *red circle* (the merge of the EGFP and transmission light channels)

Previously published experimental data indicated that knockout (or knockdown) of wild-type lamin A and its mutant ∆50 expression affects differentiated cells by slowing down their proliferation rate (Cohen et al. 2013; Vidak et al. 2015). In order to test this effect on a tissue culture model of less-differentiated cells, we used HEK 293 cells. We selected sublines expressing comparable amounts of exogenous proteins. Typical phenotype of those cells is presented in Fig. 7a.

HEK 293 sublines stably expressing EGFP and lamin A variants were analyzed for their proliferation rate in standard SRB assays (Fig. 7b). As expected, overexpression of the exogenous proteins slowed down the proliferation of HEK 293 cells. The expression of a “neutral” protein, such as EGFP, slowed down proliferation by 50 % compared to the control cells. Wild-type lamin A slowed down the proliferation rate by another 10 % comparing to EGFP (60 % compared to the control), which is statistically significant. Lamin A ∆50 expression resulted in about a 15 % proliferation slowdown compared to the control, but about a 200 % increase in the proliferation rate compared to wild-type lamin A. Lamin A D446V had a slightly higher proliferation rate (about 215 % compared to wild-type lamin A) than progerin. Proliferation slowdown compared to the control HEK 293 cell line was only about 12 %, which was not statistically significant.

These data indicate that lamin mutants affect proliferation also in less-differentiated cells. However, in contrast to mature cells (Kudlow et al. 2008; Vidak et al. 2015), ∆50 mutation has the opposite effect: there is no inhibition of proliferation.

### The NE-associated lamin A ∆50 and D446V fractions have similar mobility to wild-type lamin A

Different mutations affect lamin A properties in different ways. Since the basic properties of lamin are polymerization, filament formation, and interaction with integral membrane proteins and chromatin, the mobility analyses of mutant protein inside cells may indicate the extent of abnormality of the mutant. The most characteristic mutant from the point of view of mobility is lamin A ∆50, since it is permanently farnesylated and presumably immobilized to the NE membrane (even when not polymerized) due to the lipophilic farnesyl “anchor.”

For studies of lamin mobility, we used the FRAP technique with the established cell lines stably expressing comparable amounts of a given lamin. The mobility of all of the lamins was analyzed at the NE using EGFP as a control protein with high mobility (Fig. 7). Figure 7a shows the analyzed cell phenotypes and confirms the comparable level of expression of exogenous lamin A. Figure 7c shows the actual mobility data and detection range (insert).

The analyses revealed that lamin A and all its studied mutants (together with ∆50) showed similar low mobility during all experiments in the HEK 293 cells. Lamin A D446V showed the highest mobility (up to 10 % higher than lamin A), while lamin A ∆50 showed the lowest (about 10 % lower than lamin A). No statistical significance was detected within this trend.

## Discussion

Mutations in the *LMNA* gene are associated with over 15 disease phenotypes, including striated muscle diseases, peripheral neuropathy, lipodystrophy, bone diseases, and premature ageing syndromes with multisystemic pathology (Schreiber and Kennedy 2013). Although disease-causing mutations are located in known hot spots in some laminopathies (e.g. HGPS, mandibulacral dysplasia, familial partial lipodystrophy, and restrictive dermopathy), there are no clear genotype–phenotype correlations in other conditions (mainly those affecting skeletal and cardiac muscles). We might suspect that the complex spectrum of those diseases is associated with a disturbed development that influences particular tissues depending on the mutation type.

We investigated lamin A mutants introduced into three cell lines: HEK 293, HeLa, and NHDF. Our focus was on HEK 293 due to its interesting properties and the poor state of knowledge about lamins in less-differentiated cells. This line was previously used for analysis of lamin A mutants, but only for rough screening (Tan et al. 2015) or as an additional control for fibroblasts studies (Capanni et al. 2009; Capanni et al. 2012). The phenotypes of the other cell lines transfected with lamin A mutants were similar, but less pronounced.

HEK 293 is an immortalized embryonic cell line, with high transfection efficiency and a good tolerance of exogenous proteins. We showed that it has a very low level of lamin A and a moderate level of lamin C. Low lamin A levels confirm HEK 293 as a good model for less-differentiated cell lines (Constantinescu et al. 2006). In addition, its lamin C migrates faster in western blot than that of NHDF or HeLa cells, which suggests different posttranslational modifications or an alternative spliced form that was not detected previously. Although the mRNA level for lamin A is similar to that for lamin C, the main spliced protein form in the cell is lamin C. This can result from mRNA translation inhibition or proteolytic degradation or autophagy, or both (Cenni et al. 2011; Dou et al. 2015). Since exogenously expressed lamin can undergo autophagy-related proteolysis in HEK 293 cells (Cenni et al. 2011), and proteolytic degradation may be induced among other mechanisms by Akt1 phosphorylation of lamin A on serine 404 (Cenni et al. 2008; Bertacchini et al. 2013), it is plausible that autophagy-related proteolysis may be a possible mechanism. Similar feature of unpredicted level of expression was observed in the cause of lamin A nonsense mutant (pArg321Ter) causing dilated cardiomyopathy (Al-Saaidi et al. 2013).

HEK 293 cells are known to have the molecular properties of neural cells (Shaw 2002), and lamin A is known to be specifically downregulated within neural cells by miR-9 (Jung et al. 2012). However, miR-9 seems to be absent in HEK 293 cells (Giusti et al. 2014) but the expression of the homologous sequence of miR-79 has never been tested. In addition, we observed, beside normal proteins, faster migrating form of lamin A and mutants in western blots. This implies that in HEK 293 cells may exist posttranscriptional mechanism regulating lamin A level based on another mechanism than in neuronal cells. However, the molecular background of this mechanism needs to be resolved.

To test the effect of laminopathy-associated mutations at the cellular level, we introduced a set of lamin A mutants to less-differentiated and differentiated model cell lines. We focused on the stage before differentiation and investigated the impact of disturbing the lamin A balance and the effect of the particular mutations connected with EDMD2 and HGPS on the cell phenotype and proliferation. We chose novel mutations, L263P and D446V, which had been found in Polish patients, and we provided their molecular characterization for the first time. As a comparison, we used the better-known mutations E358K, H222P, and Δ50. Patients bearing the chosen mutations were deeply characterized clinically to allow for a better understanding of the relationship between genotype and phenotype. The phenotypes of EDMD2 patients were of comparable severity (Table 1), but the chosen mutations provided a wide spectrum of nuclear phenotypes that were significantly enhanced in HEK 293 cells compared to the differentiated cells (Figs 3, 4 and S1). The mutants differ in their tendency to aggregation, cellular toxicity, localization (NE vs. nucleoplasm) and impact on lamin C (Figs. 3 and 4). L263P has the greatest tendency to form foci, even at low expression levels. H222P also forms foci but less efficiently. E358K is translocated to the nucleoplasm and aggregates only at the highest expression levels. D446V forms lattice-like structure similar to those of wild-type lamin A in HEK 293 cells.

The different properties of these proteins may result from their location within various domains. H222P and L263P are located within or close to linkers in the coil 2A domain. They introduce the proline residue known as protein secondary structure disruptor. Those mutations are most likely to affect lamin dimerization, and the obtained results suggest that they enhance this process. E358K is located in part of the coil 2B domain that is highly conserved among vertebrates and invertebrates (Strelkov et al. 2004). It changes the amino acid residue polarity as negatively charged glutamic acid is replaced by positively charged lysine. This mutation can affect filament assembly but not dimerization (Strelkov et al. 2004). D446V is localized within the Ig fold and substitutes an acidic residue into a neutral one in an evolutionally conserved region of negatively charged amino acid residues (^442^VEEVDEEG). This amino acid was found to be exposed within the Ig fold, so it was predicted to influence DNA, RNA, and/or protein binding rather than affect protein structure (Dittmer et al. 2014; Scharner et al. 2014) or polymerization (Machowska et al. 2015). This is supported by our studies: D446V did not visibly change lamin A properties or the cellular phenotype.

The chosen set of *LMNA* gene mutations can be useful for further studies, because they probably cause EDMD2 through various mechanisms. Different aggregates formed by lamin A mutants can lead to different patterns of redistributed interacting proteins, among other effects–e.g., E358K may redistribute interacting proteins into nucleoplasm as it does for lamin C (Figs. 3 and 4) while the most toxic L263P may recruit interacting proteins (also a fraction of lamin C) into intranuclear foci (Fig. 4).

The regulation of A-type lamins during development and in particular tissues seems to be a very complex process. At least two major signaling pathways essential in development may be affected by the expression of lamin A/C. These are pRB pathway and Wnt/β-catenin pathway while retinoic acid signaling pathway, also essential for development, contributes to lamin A/C expression and is also affected by lamin A (for review, see Dubinska-Magiera et al. 2013). Lamin A is expressed in early development, then lost and expressed again during differentiation. *LMNA* promoter is activated in some tissues, e.g., the heart, liver, and somites, on day 11 of mouse embryonic development (Kubben et al. 2011). On the other hand, A-type lamins in the heart, liver, and other organs were undetectable using immunodetection after birth (Rober et al. 1989). This suggests an additional mechanism that inhibits A-type lamin expression. More recent studies revealed that low-level A-type lamins are detectable in embryonic stem cells and mouse blastocysts (Eckersley-Maslin et al. 2013). In addition to their promoter activity, A-type lamin expression is known to be regulated through alternative splicing or mRNA degradation. Lamins A and C are present at various levels in different tissues. For example, lamin A is specifically downregulated by miR-9 in brain (Jung et al. 2012).

Lamin C distribution was revealed to be dependent on lamin A level, localization, and mutations (Fig. 4). It was previously shown that lamin C and emerin targeting to the NE depends on lamin A (Sullivan 1999; Vaughan et al. 2001). Two *LMNA* spliced forms (lamin C and lamin A ∆50) even seem to have opposite functions in the context of energy metabolism (Lopez-Mejia et al. 2014). It is known that lamins A and C have different expression patterns and binding properties, and it was proposed that the imbalance between those spliced forms can result in pathogenesis (Al-Saaidi and Bross 2015). As observed in our study, lamin C redistribution may suggest the mechanism of this disturbance. In addition, emerin localization outside the nucleus (Fig. 2) can be the result of very low level of lamin A in HEK 293 cells. On the other hand, in sublines stably expressing exogenous lamin A no redistribution of emerin was observed (Fig. 6) which may suggest that interaction with lamin A is not an only mechanism responsible for emerin location (Salpingidou et al. 2007; Salpingidou et al. 2008; for review, see Berk et al. 2013). In general, via immunofluorescence, we observed that the higher the level of exogenous lamin A, the lower the level of endogenous lamin C, especially within the NE (Figs. 3 and 4). However, western blot analysis showed that the level of endogenous lamin C is stable (Fig. 5, performed on cells with at least 90 % transfection efficiency). This suggests that lamin C is not downregulated nor degradated, but dispersed within the cell. Lamin C redistribution was previously shown in X-EDMD patient cells (Markiewicz et al. 2002). Our observations of different lamin A mutants allow us to propose that the mechanism of lamin C translocation is partially based on binding by lamin A. When E358K, which localizes predominantly in nucleoplasm, is overexpressed, lamin C also localizes mostly there, while in cases of ∆50 overexpression (permanently bonded with the NE and localized there), lamin C is localized only within NE (Fig. 4).

In order to investigate the impact of novel mutations on cell proliferation and protein mobility, we established HEK 293 sublines that stably express lamin A and its mutants Δ50 and D446V. L263P mutations were found to be extremely toxic, and it was not possible to establish a subline expressing this mutant. Lamin A mutations did not change protein mobility significantly within the nuclear rim, which was not surprising, because lamin mutants rarely change it (Broers et al. 2005).

Proliferation tests revealed that lamin A overexpression leads to diminishing propagation of cells, as was previously reported for immortalized fibroblasts (Vidak et al. 2015) or COS-7 cells (Ivorra et al. 2006). Surprisingly, the presence of ∆50 or D446V in cells reversed the decrease in proliferation (Fig. 7). This suggests that HEK 293 cells may have a different (or abolished) mechanism of cell division control. To investigate this phenomenon, we performed cell cycle analysis, but it did not show any difference in the cell cycle phases between the sublines (data not shown). Cell cycle arrest was previously reported in cells obtained from *LMNA* ^*−/−*^ mice (Pekovic et al. 2007; Cohen et al. 2013), but we did not observe it here with lamin A mutants present.

It was also described that lamin A ∆50 expression is correlated with a diminishing proliferation rate in aged patient-derived or immortalized fibroblasts (Scaffidi and Misteli 2006; Kudlow et al. 2008). In addition, various lamin A mutations decrease the colony-forming capacity of mesenchymal stem cells (Malashicheva et al. 2015).

The adverse effect observed for HEK 293 cells may result from changed pathways due to immortalization with adenovirus proteins E1A and E1B (Lin et al. 2014). E1B can inhibit the p53 pathway and E1A can sequestrate to Rb, releasing and activating E2F transcription factors (Chakraborty and Tansey 2009). It was shown that in fibroblasts, ∆50-dependent diminishing of proliferation can rely on p53 (Kudlow et al. 2008) and Rb localization and activity depend on the presence of lamin A (Pekovic et al. 2007). Impairment of these pathways may result in a different effect of lamin A and its mutants on the proliferation rate in HEK 293 cells. It should be pointed out that the presence of the lamin A mutants D446V or Δ50 cause twofold faster cell propagation compared with “neutral” protein EGFP overexpression (Fig. 7). This suggests that both ∆50 and D446V may stimulate proliferation.

## Electronic supplementary material

Below is the link to the electronic supplementary material.

Figure S1 (PDF 231 kb)

Figure S2 (PDF 174 kb)
