# Supplementary material for: The effect of the lamin A and its mutants on nuclear structure, cell proliferation, protein stability, and mobility in embryonic cells
Source: Chromosoma. 2016 Aug 17;126(4):501–17. doi: 10.1007/s00412-016-0610-9 (PMC5509783; doi:10.1007/s00412-016-0610-9)

Figure S1. Analysis of NHDFs transiently transfected with EGFP–lamin A (wild-type and mutants).

(A) Immunofluorescence analysis showed that L263P protein had a speckle-like distribution that also disturbed lamin C. D446V protein had a distribution similar to wild-type lamin A. GFP tag and lamin C colocalization was noticeable.

(B) Western blot analysis of NHDF (50,000 cells per lane) showed 3 forms of lamin A/C: exogenous fusion protein EGFP–lamin A, endogenous lamin A, and endogenous lamin C. As transfection efficiency was about 20%, the comparison of endogenous and exogenous lamins is not quantitative. The L263 band was less intense than the wild-type band due to higher toxicity of this mutant (less transfected cells survived). Immunodetection was performed by Jol2 antibody (Abcam), which recognizes the epitope in D446. Thus, mutation D446V impairs binding with the antibody.

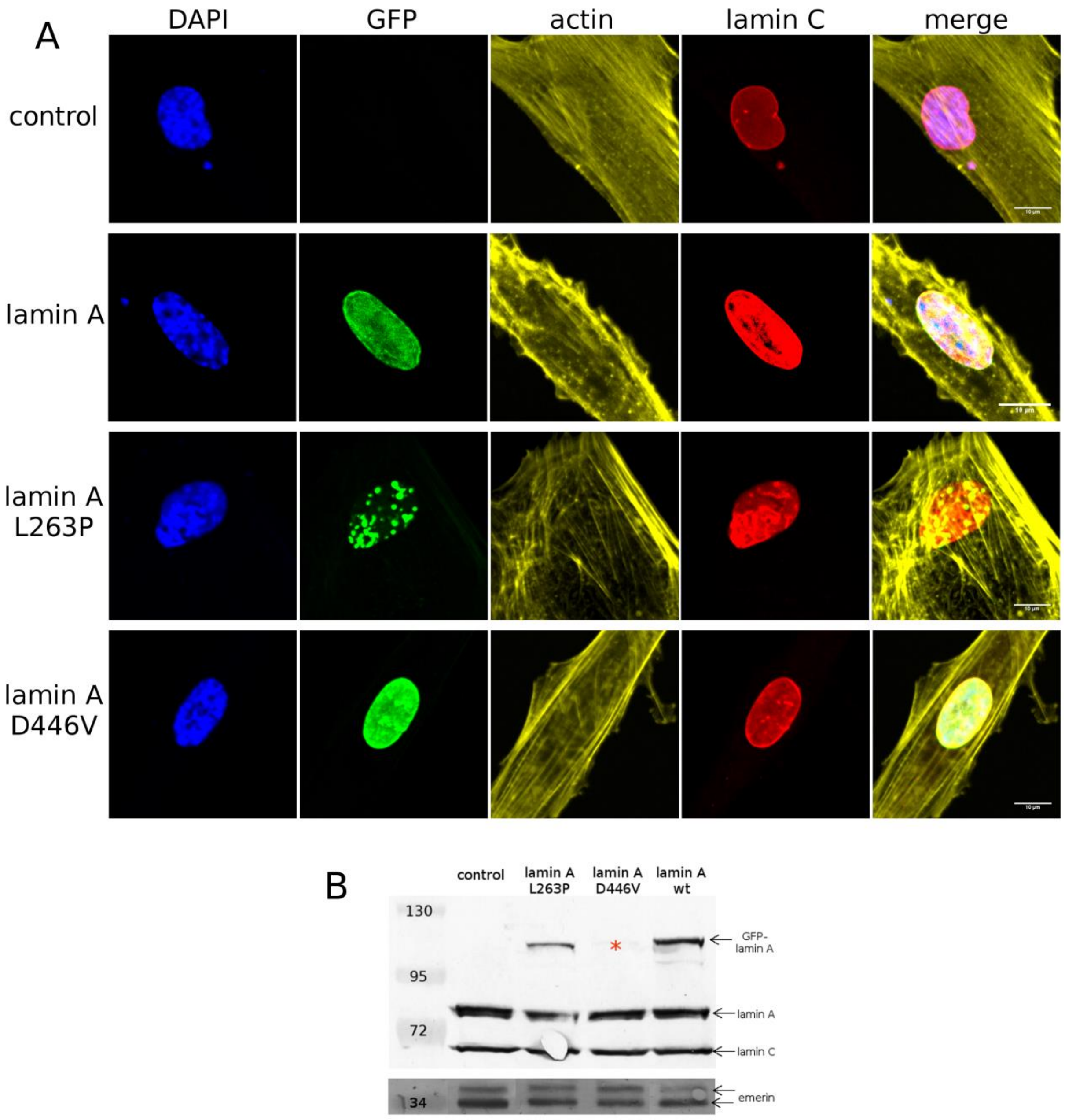

Supplement: Supplementary file 1 — (PDF 231 kb) [file 412_2016_610_MOESM1_ESM.pdf]
