# Supplementary material for: The effect of the lamin A and its mutants on nuclear structure, cell proliferation, protein stability, and mobility in embryonic cells
Source: Chromosoma. 2016 Aug 17;126(4):501–17. doi: 10.1007/s00412-016-0610-9 (PMC5509783; doi:10.1007/s00412-016-0610-9)

**Figure S2.** Z-stacking reconstruction of typical nuclei of transfected HEK 293 cells shows differences between protein localization for wild-type lamin A and its mutants D446V and Δ50 (progerin).

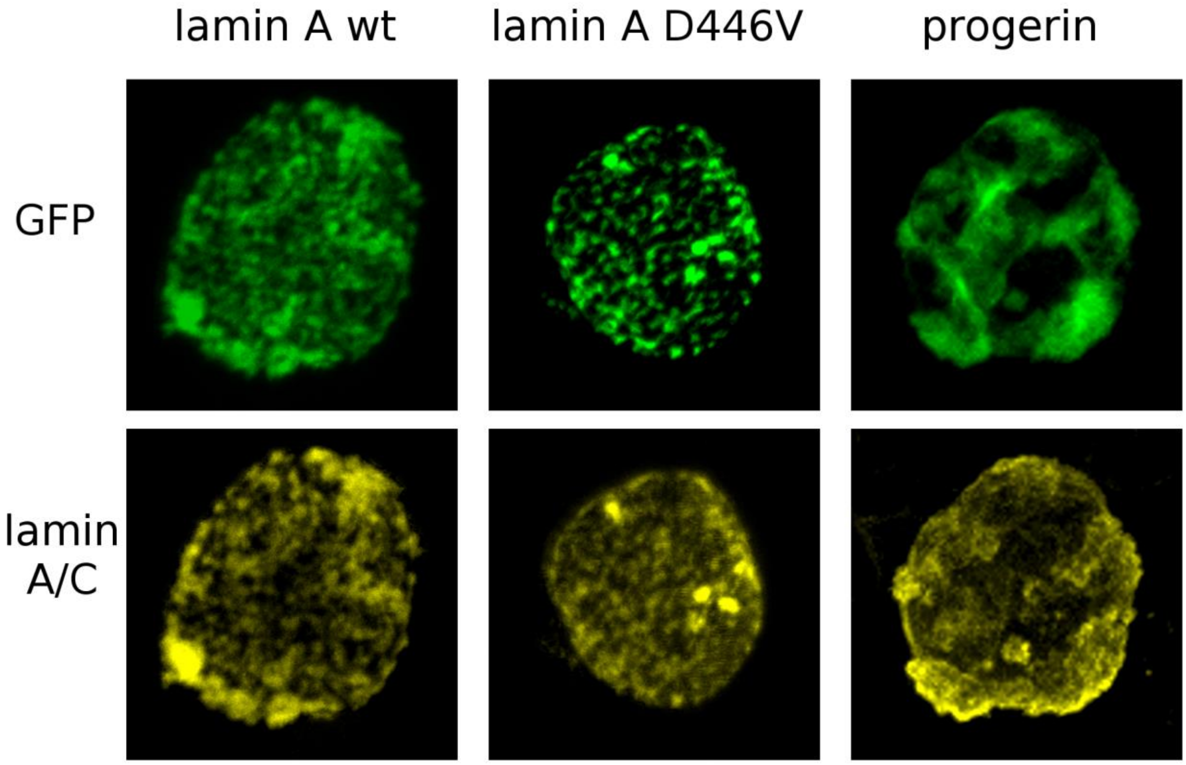

Supplement: Supplementary file 2 — (PDF 174 kb) [file 412_2016_610_MOESM2_ESM.pdf]
